# New positive feedback mechanism between boundary layer meteorology and secondary aerosol formation during severe haze events

**DOI:** 10.1038/s41598-018-24366-3

**Published:** 2018-04-17

**Authors:** Quan Liu, Xingcan Jia, Jiannong Quan, Jiayun Li, Xia Li, Yongxue Wu, Dan Chen, Zifa Wang, Yangang Liu

**Affiliations:** 1 Beijing Weather Modification Office, Beijing, China; 2 Institute of Urban Meteorology, Chinese Meteorological Administration, Beijing, China; 3 Brookhaven National Laboratory, Upton, NY 11973 USA; 4 College of Atmospheric sciences, Lanzhou University, Lanzhou, China; 5 Beijing Meteorological Bureau, Beijing, China; 6 Institute of Atmospheric Physics, Chinese Academy of Sciences, Beijing, China

## Abstract

Severe haze events during which particulate matter (PM) increases quickly from tens to hundreds of microgram per cubic meter in 1–2 days frequently occur in China. Although it has been known that PM is influenced by complex interplays among emissions, meteorology, and physical and chemical processes, specific mechanisms remain elusive. Here, a new positive feedback mechanism between planetary boundary layer (PBL), relative humidity (RH), and secondary PM (SPM) formation is proposed based on a comprehensive field experiment and model simulation. The decreased PBL associated with increased PM increases RH by weakening the vertical transport of water vapor; the increased RH in turn enhances the SPM formation through heterogeneous aqueous reactions, which further enhances PM, weakens solar radiation, and decreases PBL height. This positive feedback, together with the PM-Radiation-PBL feedback, constitutes a key mechanism that links PM, radiation, PBL properties (e.g. PBL height and RH), and SPM formation, This mechanism is self-amplifying, leading to faster PM production, accumulation, and more severe haze pollution.

## Introduction

China has been experiencing heavy air pollution in the past two decades, with frequent severe haze events^1,2^. During haze events, the mass concentration of fine particulate matter (smaller than 2.5 μm in aerodynamic diameter, PM_2.5_) can reach as high as 600 μg m^−3 ^^3–6^. Besides, the increase of PM_2.5_ during haze events is steep, sometime by an order of magnitude in 1–2 days^3,7,8^. High emissions of atmospheric pollutants, including primary aerosols and their gas precursors, are thought as main causes of heavy air pollution in China^5,7,9^. Weakened atmospheric diffusion has also been considered to enhance the PM_2.5_ accumulation because stable meteorological conditions favor the accumulation of primary and secondary pollutants^10^. Furthermore, the complex interplays among emissions, PBL meteorology, and atmospheric chemical processes make it more difficult to understand the formation of severe haze events^11–15^. A key player in the web of interplays is the PBL, which constitutes the lowest atmospheric layer. There is generally a barrier (very low turbulent mixing rate) at the top of the PBL to prevent particles being transported from the PBL to the free troposphere^16,17^. As a result, aerosol particles are mainly constrained in the PBL, and aerosol concentrations are anti-correlated with the PBL heights^11,17^. Furthermore, aerosol particles also influence the PBL stability^18–20^ and PBL height^12,13^ by decreasing the solar radiation reaching the Earth’s surface^3,11,21^ and turbulent mixing^14^. It has been recently found that aerosol particles can also affect the formation of secondary aerosols by changing photolysis rate and O_3_ formation^22,23^, and the involvement of heterogeneous aqueous reactions might make it more complex^24–28^. Despite these studies and some general understanding, physical-chemical mechanisms underlying the complex interplays remained largely elusive in such heavily-polluted environments as those encountered in China.

During the 2014 Asia-Pacific Economic Cooperation (APEC) summit, a series of emission control measures^8,29^ was implemented during 2–12 Nov. 2014 to ensure good air quality, providing a unique opportunity to understand the effect of emission reduction and the complex interplays between aerosols, meteorology (wind, radiation, PBL, and RH), and atmospheric chemical processes. We capitalized on this opportunity by organizing a comprehensive field campaign in the urban area of Beijing. A number of quantities, including atmospheric visibility, PM_2.5_ mass concentration, chemical composition of non-refractory submicron particles (NR-PM_1_), and PBL structure, were measured simultaneously, together with key meteorological variables (temperature, RH, pressure, wind, and solar radiation). This paper analyzes the data and presents the major findings from this campaign.

## Results and Discussion

**Figure 1:**
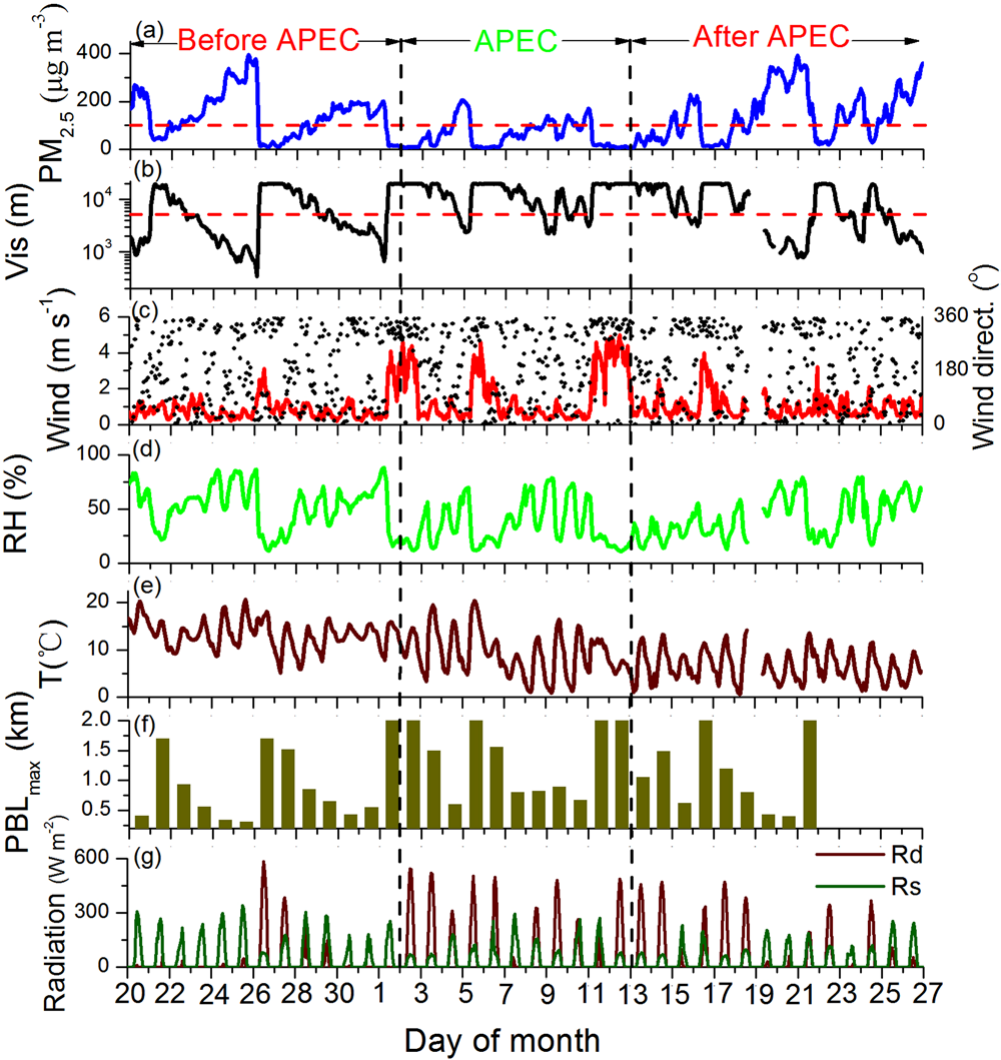
Temporal variations of PM_2.5_ mass concentration (**a**), visibility (**b**), wind (**c**), relative humidity (RH) (**d**), temperature (**e**), daily maximum PBL height (PBL_max_), averaged with data between 13:00–15:00 (**f**), and direct and scattered solar radiation (**g**). The temporal resolution of variables is 1 h, except for the PBL.

The comprehensive field campaign was conducted from 20 Oct. to 26 Nov. 2014 at the Baolian (BL) meteorological station, China Meteorological Administration (CMA) (39°56′N, 116°17′E). For comparative analysis, the period of 2–12 Nov., when the strict emission control was imposed, is chosen to represent the APEC period; whereas the periods before (20 Oct.–1 Nov.) and after (13–26 Nov.) the APEC are used to represent the non-APEC period. A total of 7 haze events (2 during the APEC and 5 during the non-APEC periods) occurred during the field campaign (haze events are defined as visibility being less than 5 km and RH less than 90%). Fig. 1 shows the temporal variations of the key variables during the whole field campaign. The PM_2.5_ concentration (Fig. 1a) and visibility (Fig. 1b) exhibited a clear cycle of 4–7 days. Before each haze episode, strong northwest wind brought clear air to Beijing with abrupt decrease of aerosols and increase of visibility. After that, wind decreased and pollutants began to accumulate until the arrival of next clean northwest air at the end of the haze event (Fig. 1c). The peak PM_2.5_ concentration reached 150–400 μg m^−3^ (Fig. 1a) and visibility decreased to 1–3 km correspondingly (Fig. 1b). There were concurrent co-variations of other key variables during the haze events. For example, RH increased (Fig. 1d); the surface direct solar radiation (R_d_) and total surface solar radiation decreased whereas the diffuse solar radiation (R_s_) increased (Fig. 1g). The increased RH further decreased visibility by enhancing hygroscopic growth of aerosol particles^30,31^.

### High PM reduces PBL height

**Figure 2:**
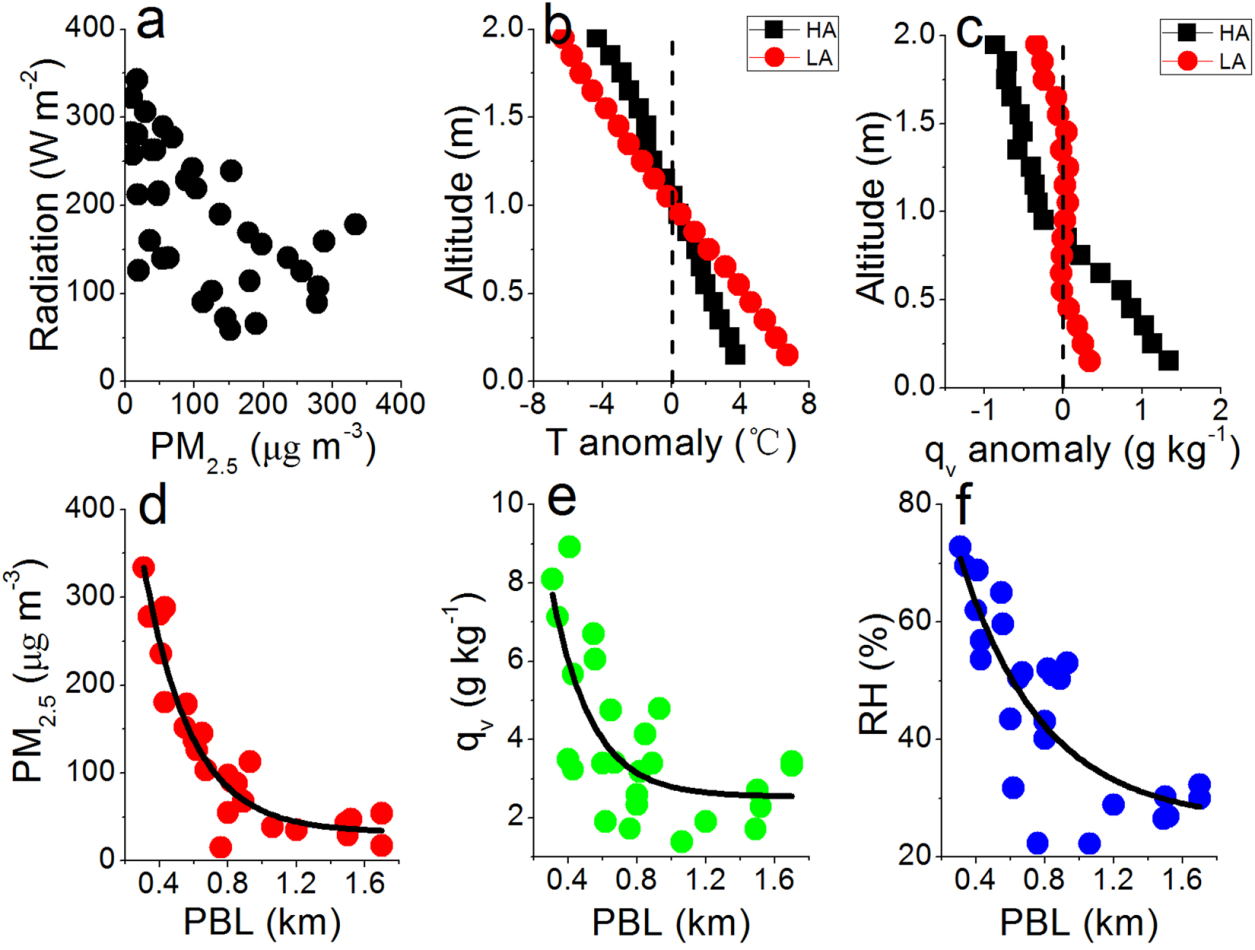
Relationships of radiation to PM_2.5_ (**a**), profiles of temperature anomaly (**b**) and water vapor maxing ratio (q_v_) anomaly (**c**) under high (HA, PM_2.5_ ≥ 75 μg cm^−3^) and low aerosols (LA, PM_2.5_ < 75 μg cm^−3^), and relationships of PM_2.5_ (**d**), q_v_ (**e**), RH (**f**) to PBL.

Aerosol particles modify radiative fluxes by scattering and absorption of solar radiation and, to a lesser extent, by absorption and emission of thermal infrared radiation^32^. Observations indicate that surface total solar radiation decreases from 243 W m^−2^ to 149 W m^−2^, with a decrease rate of 39%, when PM_2.5_ increases from <75 μg cm^−3^ (40±17 μg cm^−3^) to ≥75 μg cm^−3^ (187±81 μg cm^−3^) (Fig. 2a), which is consistent with the calculated aerosol radiative forcing over North China Plain^13^. To see the radiative effect of aerosols on temperature profile, the sounding data are partitioned into two groups of high (HA) and low (LA) aerosols (Fig. 2b,c). The HA and LA groups represent the averages of all samples with the ground PM_2.5_ ≥75 μg cm^−3^ and PM_2.5_ <75 μg cm^−3^, respectively. A comparison of the temperature profiles reveals that low layer (<1 km) temperature decreased by 3.2 °C under HA while high layer (>1 km) temperature increased by 2.4 °C, suggesting that atmosphere become more stable under high aerosols. A decreased downwelling direct solar radiation, together with stabilized atmosphere, could reduce turbulence kinetic energy^11,33^ under high aerosols. The latter is the dominant factor that influences the development of the planetary boundary layer (PBL). As a result, the increased aerosols reduce PBL height. The daily maximum PBL height (PBL_max_), averaged with data between 13:00–15:00, decreased from 1.3 to 0.6 km when ground PM_2.5_ increased from < 75 μg cm^−3^ to ≥ 75 μg cm^−3^.

### Suppressed PBL enhances RH

There is generally a barrier (very low turbulent mixing rate) at the top of the PBL to prevent pollutants and water vapor being transported across from the PBL to the free troposphere^16,17^. As a result, aerosol particles, which origin mainly from ground emission, are mainly constrained in the PBL and aerosol concentration is anti-correlated with PBL height (Fig. 2d). Similar as aerosol particles, a large part of atmospheric water vapor inside PBL comes from surface evaporation^34^ and anthropogenic emissions^35^, especially under stable condition when haze event occurs. Hence, water vapor mixing ratio (q_v_) is also anti-correlated with PBL height (Fig. 2e). To further verify this point, q_v_ profiles are analyzed (Fig. 2c). The vertical q_v_ gradient under high aerosols is much higher than under low aerosol, suggests that vertical transportation is suppressed and thus water vapor is accumulated in the lower layer (<1 km). The RH is closely related to water vapor; a high water vapor content corresponds to a high relative humidity under certain temperature. Therefore, the suppressed PBL caused by high aerosols increases RH inside PBL (Fig. 2f).

The PM_2.5_ concentration, q_v_, and RH increased with decreased PBL height, and their relationships appear non-linear. When PBL height was high, the change in PM_2.5_, q_v_ and RH were weakly sensitive to PBL height. In contrast, when PBL height is low, the change in PM_2.5_, q_v_ and RH were strongly sensitive to PBL height. The transition point corresponds to the PBL height of 0.8 km. For example, when PBL_max_ decreased from 1.6 km to 0.8 km, PM_2.5_ increased from 38 μg cm^−3^ to 75 μg cm^−3^. The ratio of the changes between PM_2.5_ and PBL height (ΔPM_2.5_/ΔPBL_max_) was −46 (μg cm^−3^ km^−1^). In contrast, when PBL_max_ decreased from 0.8 km to 0.4 km, PM_2.5_ increased from 75 μg cm^−3^ to 250 μg cm^−3^. The ratio of (ΔPM_2.5_/ΔPBL_max_) is −470 (μg cm^−3^ km^−1^), which was about 10 times higher than the first value. The positive feedback of aerosol-PBL might act as a plausible explanation for above phenomenon. Based on the work of Petäjä^14^, this feedback remains moderate at lower PM loadings, but becomes increasingly effective at higher PM loadings. High aerosols change the vertical temperature profile, cause the decay of turbulence and lowering of the PBL height. In turn, a direct consequence of the lowered PBL is increases in aerosols and water vapor since emissions were confined to a smaller volume. Therefore, in severe haze events with high aerosols, the positive feedback of aerosols-PBL further accelerates the accumulation of aerosol particles and water vapor inside PBL.

### Enhanced RH accelerates SPM formation

**Figure 3:**
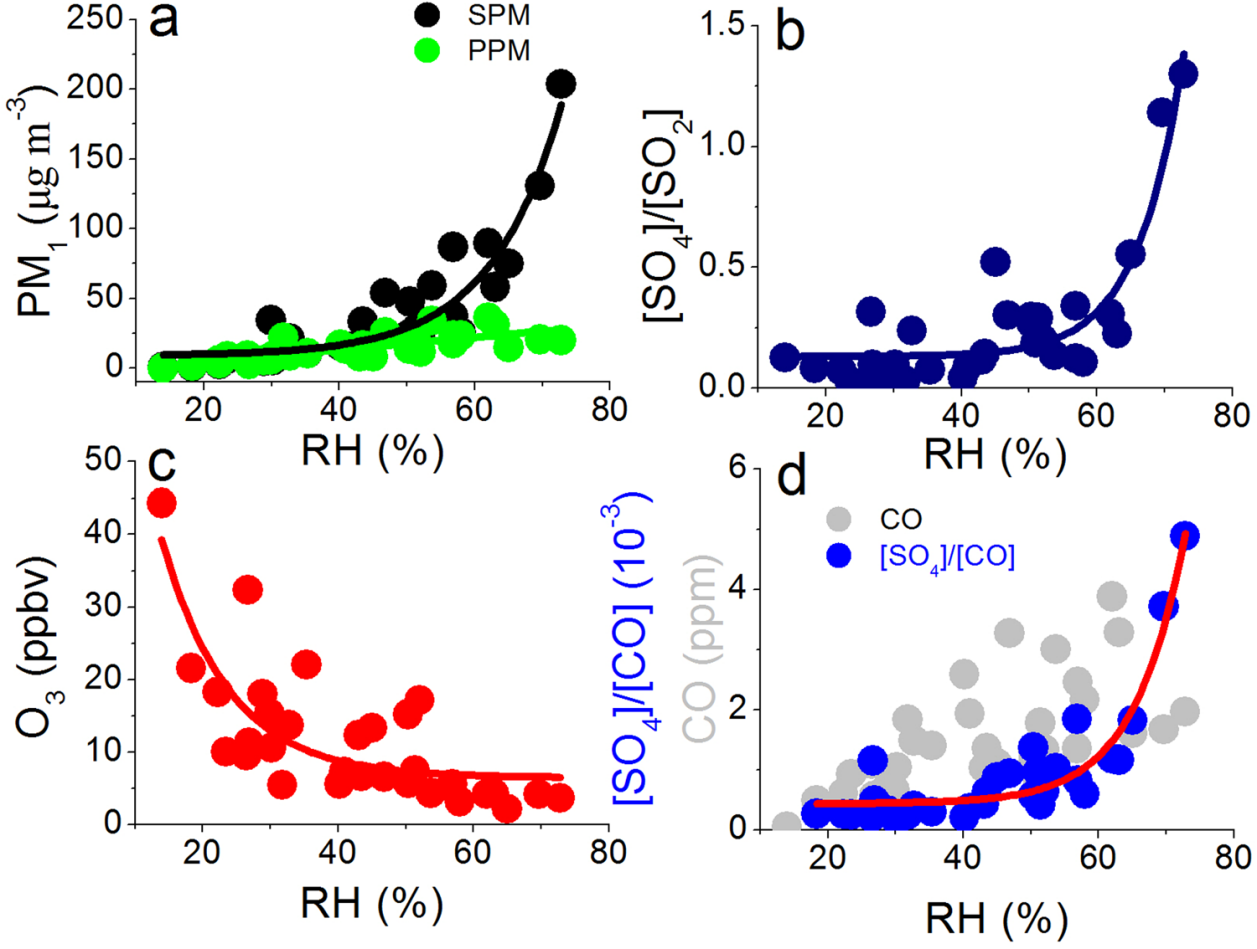
Relationships between RH and SPM and PPM (**a**), [SO_4_]/[SO_2_] (**b**), O_3_ (**c**), and [SO_4_]/[CO] and CO (**d**).

Secondary particles, including sulfate (SO_4_), nitrate (NO_3_), ammonium (NH_4_), and secondary organic aerosols (SOA) etc., contribute a large portion of PM_2.5_ during severe haze events^3,6–8^. The conversion of gas precursors to particles is related to complex reaction mechanisms, including photochemistry reactions, heterogeneous aqueous reactions, and even cloud reactions^28^. For heterogeneous aqueous reactions, they are strongly related to RH. Under high RH, some aerosol particles likely experience hygroscopic growth, and their radii can be doubled, or approximate 8 times increase of particle volume^31^, which provides an excellent substrate for heterogeneous aqueous reactions. To understand the contribution of heterogeneous aqueous reactions in haze events, relationships between RH and mass concentration of secondary PM (SPM), primary PM (PPM), and the sulfate production rate are analyzed (Fig. 3). The concentration ratios of sulfate to sulfur dioxide ([SO_4_]/[SO_2_]) is used as a proxy for the sulfate production rate^10,28^. The observations showed that the PPM concentration increased with RH in a moderate rate while the increase of SPM with RH is much more dramatic, especially when RH is higher than 60% (Fig. 3a), indicating accelerated SPM formation under high RH. For example, when RH increases from 50–60% to 60–70% the SPM concentration increases from 40 μg m^−3^ to 88 μg m^−3^, with an increase rate of 123%, about 4 factor of higher than the PPM increase (from 19 μg m^−3^ to 26 μg m^−3^, with an increase rate of 32%). The variation of sulfate production rate with RH further confirmed this result; The ratio ([SO_4_]/[SO_2_]) increases significantly when RH is higher than 60% (Fig. 3b), which is consistent with SPM variation. In contrast, the O_3_ concentration is significantly reduced in this process, indicating a decrease of photochemistry capacity (Fig. 3c). The reduced photochemistry capacity and increased SPM production suggest the important contribution of heterogeneous aqueous reactions in secondary particles formation during severe haze events.

In addition to chemical processes, aerosol mass concentration is influenced simultaneously by meteorological processes. To estimate the relative contribution of chemical processes in the haze events, sulfate-CO ratio ([SO_4_]/[CO]) is calculated. In this calculation, CO is selected as a inactive chemical tracer because of its long chemical lifetime. The chemical lifetime of CO is about a few months^36^. As a result, the variability of CO is mainly controlled by meteorological factors and sulfate-CO ratio can be estimated approximatively as the contribution of chemical processes^10^. As we can see from Fig. 3d that CO concentration increased with RH, suggests that they were affected simultaneously by the weakening atmospheric diffusion capacity in haze events. In addition, When RH was lower than 40%, [SO_4_]/[CO] kept nearly constant, but increased with further increasing RH especially when RH is higher than 60%, in consistence with the trend of sulfate production rate as a function of RH. Aerosol particles begin hygroscopic growth around 40% based on previous studies^37^. After that, the hygroscopic growth factor increased exponentially. The consistence of sulfate production rate to RH with aerosol hygroscopic growth further supports the role of heterogeneous aqueous reactions in SPM formation. Therefore, it is safe to conclude that heterogeneous aqueous reactions play dominant contribution in the formation of secondary particle formation during severe haze events; an increase of RH helps to accelerate the conversion of gas precursors to particles.

### Feedback mechanism of PBL-RH-SPM

Based on above analysis, the low PBL height helps to enhance water vapor and hence RH inside PBL, which in turn enhances the conversion of gas precursors to particles through heterogeneous aqueous reactions, further increasing PM concentration. The increased PM could further decrease PBL height due to the positive feedback of PM-radiation-PBL^11,12,14^. Furthermore, increased RH induces hygroscopic growth and an increase of aerosol particle size^30,31^, which magnifies their effects on radiation and further decrease of PBL height. These results collectively suggest a plausible positive PBL-RH-SPM-PM feedback mechanism in haze events. This feedback mechanism, together with the positive feedback mechanism of PM-radiation-PBL^11,12,14^, constitute a comprehensive positive loop of feedback mechanisms that links PM, radiation, PBL properties (e.g. PBL height and RH), and SPM formation, which could rapidly enhance PM in severe haze events.

According to this feedback loop, the formation of severe haze events can be summarized as follows: in the beginning of haze events, PM concentration and RH are low, and daytime PBL height is high. Weakened transport triggered by stagnant weather conditions leads to the PM accumulation. Increased PM suppresses the development of PBL by weakening solar radiation, leading to lower PBL height. Suppressed PBL helps to increase PM and RH, which induces hygroscopic growth of aerosol particles, further magnifies their effect on radiation and decrease of PBL height. Increased RH further enhances the formation of SPM through heterogeneous aqueous reactions.

The emission control during the APEC period provides a unique opportunity to evaluate the proposed feedback mechanisms. The mean APEC PM_2.5_ concentration was 61 μg m^−3^, decreased by 58% compared to that during the non-APEC period (144 μg m^−3^, figure S1(a)). The decrease of PM_2.5_ during the APEC period led to the corresponding increases of solar radiation (47%, figure S1(c)) and hence PBL_max_ height, which increased to 1.35 km during the APEC relative to 0.99 km in the non-APEC correspondingly (figure S1(d)). In the haze events, PBL_max_ decreased from 2.0 to 0.6–0.8 km during the APEC, as compared to 0.3–0.4 km during the non-APEC. The averaged RH decreased from 49% in the non-APEC to 37% in the APEC (figure S1(d)). The decrease in RH during the APEC slowed down the transfer of precursors to SPM. The fraction of SPM in NR-PM_1_ decreased from 84% before the APEC to 62% in the APEC period (figure S2), indicating a slowdown of the chemical conversion of gaseous precursors to aerosols and helped to further decrease the PM_2.5_ concentration in the APEC.

### Model simulation

**Figure 4:**
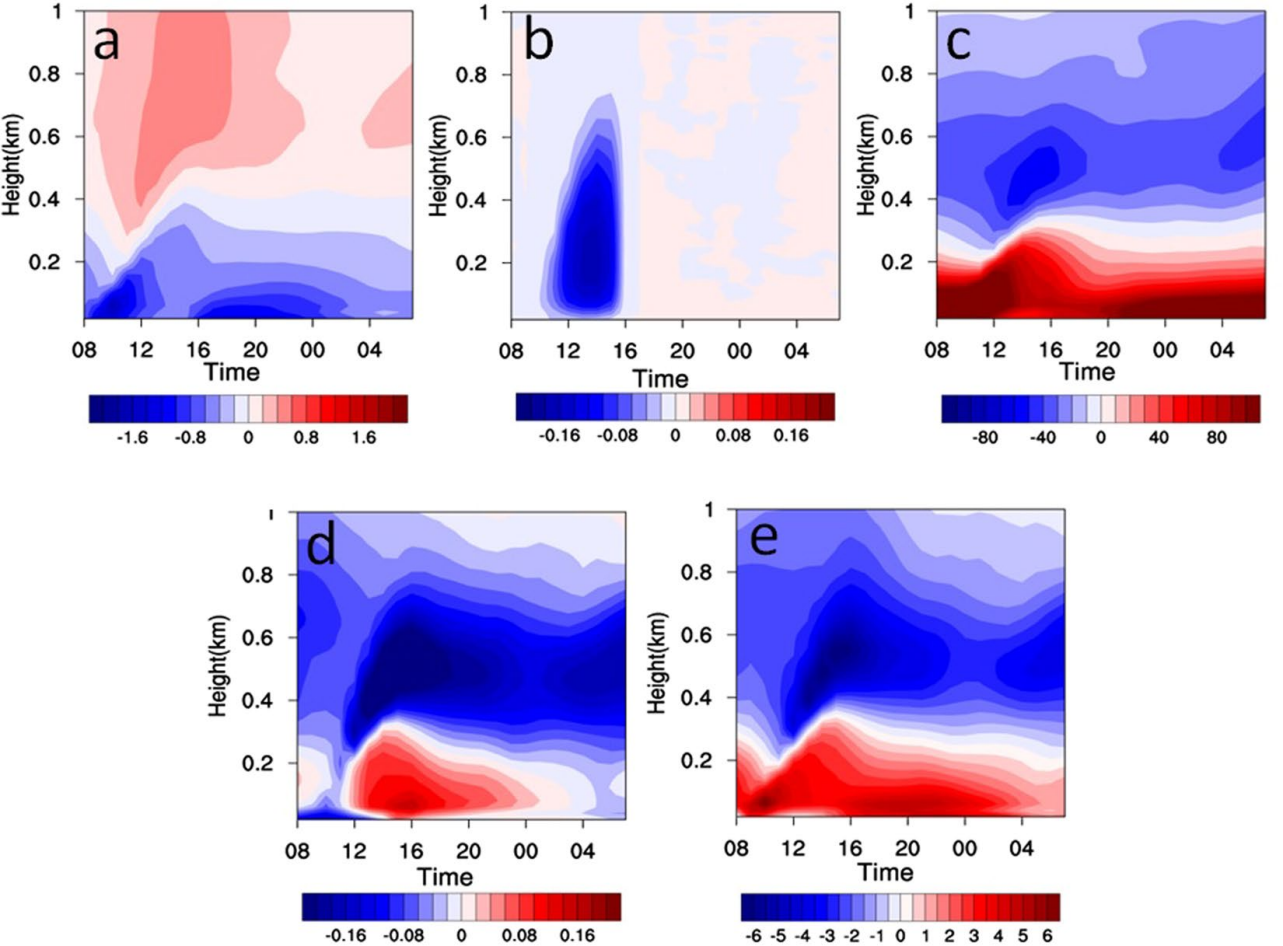
Vertical distribution of aerosol-radiation feedback induced diurnal difference (Scenario-4 - Scenario-3) in temperature (**a**, °C), TKE (**b**, J kg^−1^), PM_2.5_ concentration (**c**, μg m^−3^), water vapor content (**d**, g kg^−1^), and RH (**e**, %) for the study region during 18 to 21 December, 2016.

To further understand the feedback mechanisms and quantitatively estimate their contributions, model simulations are conducted with WRF-Chem^38^. The aerosol radiation effect, photochemistry, and heterogeneous reactions are considered, among other processes, in the simulations. Briefly, four simulations were conducted to investigate aerosols radiation (AR) effect and heterogeneous reactions. The analysis of the simulations shows that AR effect cools the lower PBL while warms the upper PBL (Fig. 4a), enhancing atmospheric stability. In this process, the turbulence kinetic energy (TKE) in the lower PBL also decreases (Fig. 4b). The combined thermodynamic and dynamic variations caused by AR effect suppress the PBL development and its height decreases correspondingly (Fig. S3). The decreased PBL height (PBLH) weakens the vertical diffusion of aerosols, resulted in more aerosols in the lower atmosphere (Fig. 4c). Similar as aerosols, the decreased PBLH also alters the water vapor profile and more water vapor is accumulated in the lower atmosphere (Fig. 4d). The combined role of increased water vapor and decreased temperature result in higher RH in the lower atmosphere (Fig. 4e). The increased RH will promote the aerosol heterogeneous reactions and increase aerosol concentration correspondingly, which further enhances the aerosol radiation effect.

To quantitatively identify the contributions of AR effect and heterogeneous reactions, some key variables in the four simulations are compared, including shortwave radiation (SW), temperature, TKE, PBLH, RH, and PM_2.5_ (Fig. S3). When AR effect is included (Scenario 2 - Scenario 1), the SW, temperature, TKE, and PBLH decrease by 69 W m^−2^, 0.7 °C, 0.01 J kg^−1^, and 147 m, respectively, while RH and PM_2.5_ increased by 3% and 56 μg m^−3^ respectively. When heterogeneous reactions are considered (Scenario 3 - Scenario 1), the PM_2.5_ increases by 63 μg m^−3^. When both AR effect and heterogeneous reactions are included simultaneously (Scenario 4 - Scenario 1), PM_2.5_ increases by 179 μg m^−3^. Several points are noteworthy from the simulations. First, the simulated PM_2.5_ concentration is consistent with the observation when both AR effect and heterogeneous reactions are included, which are 275 μg m^−3^ (observation) and 310 μg m^−3^ (simulation) respectively, while PM_2.5_ concentration is only 139 μg m^−3^ when neither AR effect nor heterogeneous reactions is included (Scenario 1), much lower than the observation, indicating the important roles of AR effect and heterogeneous reactions on PM_2.5_ in heavy air pollution events. Second, the PM_2.5_ increase (179 μg m^−3^) when both AR effect and heterogeneous reactions are included (Scenario 4) is higher than the sum of PM_2.5_ increase of individual AR effect (56 μg m^−3^) and heterogeneous reactions (63 μg m^−3^) are included, further supporting the significant role of the positive feedback mechanism proposed above.

## Conclusion

A positive feedback mechanism between boundary layer meteorology and secondary particle formation is proposed based on the observational results. It is shown that in haze events the decreased PBL increases RH by weakening the diffusion of water vapor; the increased RH in turn enhances the SPM formation through heterogeneous aqueous reactions, which further increases PM. This feedback mechanism, together with the positive feedback mechanism of PM-radiation-PBL^11,12,14^, constitutes a comprehensive positive loop of feedback mechanisms that links PM, radiation, PBL properties (e.g. PBL height and RH), and SPM formation, rapidly enhance PM in severe haze events: more PM leads to lower solar radiation, lower PBL height, higher RH, higher SPM formation, still higher PM and RH, and so on. For the same reason, reducing emissions will likely accelerate the decrease of the surface aerosol concentration. The new PBL-RH-SPM feedback mechanism, which has been largely ignored, should be used to improve air quality models, especially in heavy aerosol-polluted regions such as North China Plain.

## Methods

### Sampling and Observations

The comprehensive field campaign was conducted at the Baolian (BL) meteorological station, China Meteorological Administration (CMA) (39°56′N, 116°17′E). The measurements were taken from 20 Oct. to 26 Nov. 2014. The PM_2.5_ mass concentration was measured with a R&P model 1400a Tapered Element Oscillating Microbalance (TEOM, Thermo Scientific Co., USA) instrument, with a 2.5 μm cyclone inlet and an inlet humidity control system. Atmospheric visibility was measured with a PWD20 (Vaisala Co., Finland), and meteorology variables were measured with WXT-510 (Vaisala Co., Finland). Solar radiations, including direct, scattered, and total radiation, were measured with TB-2-B series instruments (Huatron CO., China) that covers the wavelength of 0.3–3μm. A micro-pulse lidar (MPL-4B, Sigmaspace Co., USA) was employed to study the evolution of PBL. The PBL height is determined at the altitude where a sudden decrease in the scattering coefficient occurs^39–41^. A range of gaseous species including CO, O_3_, NOx, and SO_2_ were measured by TSI instruments (TSI Co., USA). The chemical composition of NR-PM_1_ was measured with an Aerodyne compact Time-of-Flight Aerosol Mass Spectrometer (C-ToF-AMS).

### AMS Data Analysis

The AMS data were analyzed for the mass concentrations and composition with the standard ToF-AMS data analysis software package (SQUIRREL version 1.52)^42,43^. A collection efficiency (CE) factor of 0.5 was introduced to account for the particle loss, mostly due to particle bounce at the vaporizer^44,45^. The values of relative ionization efficiency (RIE) used in this study were 1.2 for sulfate, 1.1 for nitrate, 1.3 for chloride and 1.4 for organics^45^. The RIE value of 4.0 was used for ammonium based on the analysis of pure NH_4_NO_3_ particles. The PMF (Positive Matrix Factorization) analysis^46^ was performed on the organics mass spectra (m/z 12–300) following the procedures described in Ulbrich *et al*.^47^. In this study, organic aerosols were differentiated into four components by PMF analysis, including hydrocarbon-like (HOA), cooking-related (COA), coal combustion (CCOA) and oxygenated (OOA) organic aerosols. HOA, COA and CCOA are used to represent primary organic aerosols (POA), and OOA is used to represent secondary organic aerosols (SOA). In this work, Chl and POA are used to represent primary PM (PPM); SO_4_, NO_3_, NH_4_, and SOA are used to represent secondary PM (SPM).

### Model Configurations

The Weather Research and Forecasting (WRF) model coupled with online chemistry (WRF-Chem) is based upon the non-hydrostatic WRF community model (http://www.wrf-model.org/index.php). Details of the WRF-Chem model are described by Grell *et al*.^38^. We used version 3.6.1 in this study. The Morrison microphysical scheme^48^ and the Grell–Devenyi ensemble cumulus parameterization^49^ are used. The Noah parameterization is used to represent land surface processes and MYJ scheme to represent boundary layer turbulent mixing. The aerosol-radiation interaction is simulated with the Rapid Radiative Transfer Model (RRTMG)^50^ for both SW and LW radiation as implemented by Zhao *et al*.^51^. The Carbon Bond Mechanism version Z (CBMZ)^52^ and Model for Simulating Aerosol Interactions and Chemistry (MOSAIC)^53^ are used as the gas-phase and aerosol chemical mechanisms, respectively. MOSAIC uses a sectional approach to represent the aerosol size distribution with 4 and 8 size bins available in the public version of the code. In this study, we use 4 size bins with aerosols diameters ranging from 0.039–0.1, 0.1–1, 1–2.5, and 2.5–10 µm. The Fast-J photolysis scheme is used for photolytic rate calculations. New parameterizations of SO_2_-to-H_2_SO_4_ and NO_2_/NO_3_-to-HNO_3_ heterogeneous reaction rates that depend on relative humidity are applied^54^. The meteorological initial and boundary conditions are from the NCEP final analysis data (GFS). The lateral boundary conditions for chemistry and aerosol fields are based on the prescribed idealized profiles. The model domain, with 9 km horizontal grid spacing covers the North China Plain (223 × 202 grids), covers the urban clusters of northern and eastern China. There are 40 vertical layers from the surface to 50 hPa, with 10 layers below 500 m in order to simulate the haze event well. The simulation is from 15 Dec. to 21 Dec., 2016 and the first 3 days are treated as a spin-up period and are not used in analyses. Four scenarios of simulations are conducted to investigate the effects of aerosol radiation and heterogeneous reactions (see Table S1). In Scenario 1, no aerosol radiation and no heterogeneous reactions are included; In Scenario 2 aerosol radiation is included, together with the aerosol-radiation feedbacks. Scenario 3 only heterogeneous reactions; Scenario 4 considers both aerosol radiation and heterogeneous reactions. The emission inventory used in the four scenarios is the same. The differences between the four scenarios are used to isolate aerosols radiation effect and heterogeneous reactions effect.

## Electronic supplementary material

Supplementary Information
