# Supplementary material for: New positive feedback mechanism between boundary layer meteorology and secondary aerosol formation during severe haze events
Source: Sci Rep. 2018 Apr 17;8:6095. doi: 10.1038/s41598-018-24366-3 (PMC5904139; doi:10.1038/s41598-018-24366-3)
Supplement: Supplementary file 1 — Supplementary Information [file 41598_2018_24366_MOESM1_ESM.doc]

**Supplementary Information to "New positive feedback mechanism between boundary layer meteorology and secondary aerosol formation during severe haze events"**

Quan Liu^1,2†^, Xingcan Jia^2†^, Jiannong Quan^2,3*^, Jiayun Li^4^, Xia Li^1^,

Yongxue Wu^5^, Dan Chen^2^, Zifa Wang^6*^, and Yangang Liu^3*^

^1^Beijing Weather Modification Office, Beijing, China

^2^Institute of Urban Meteorology, Chinese Meteorological Administration, Beijing, China

^3^Brookhaven National Laboratory, Upton, NY 11973, USA

^4^College of Atmospheric sciences, Lanzhou University

^5^Beijing Meteorological Bureau, Beijing, China

^6^Institute of Atmospheric Physics, Chinese Academy of Sciences, Beijing, China

(21 Dec., 2017)

†These authors contributed equally to this work

Corresponding Authors

Jiannong Quan

Institute of Urban Meteorology, Chinese Meteorological Administration, Beijing, China

jn[quan@ium.cn](mailto:quanjn1975@gmail.com)

Yangang Liu

Brookhaven National Laboratory, Upton, NY 11973, USA

lyg@bnl.gov

Zifa Wang

Institute of Atmospheric Physics, Chinese Academy of Sciences, Beijing, China

zifawang@mail.iap.ac.cn

**Tables and figures**

Table S1 Simulation description

| Sensitive simulations | Aerosol radiation  feedbacks | Heterogeneous  reactions |
| --- | --- | --- |
| Scenario 1 | off | off |
| Scenario 2 | on | off |
| Scenario 3 | off | on |
| Scenario 4 | on | on |


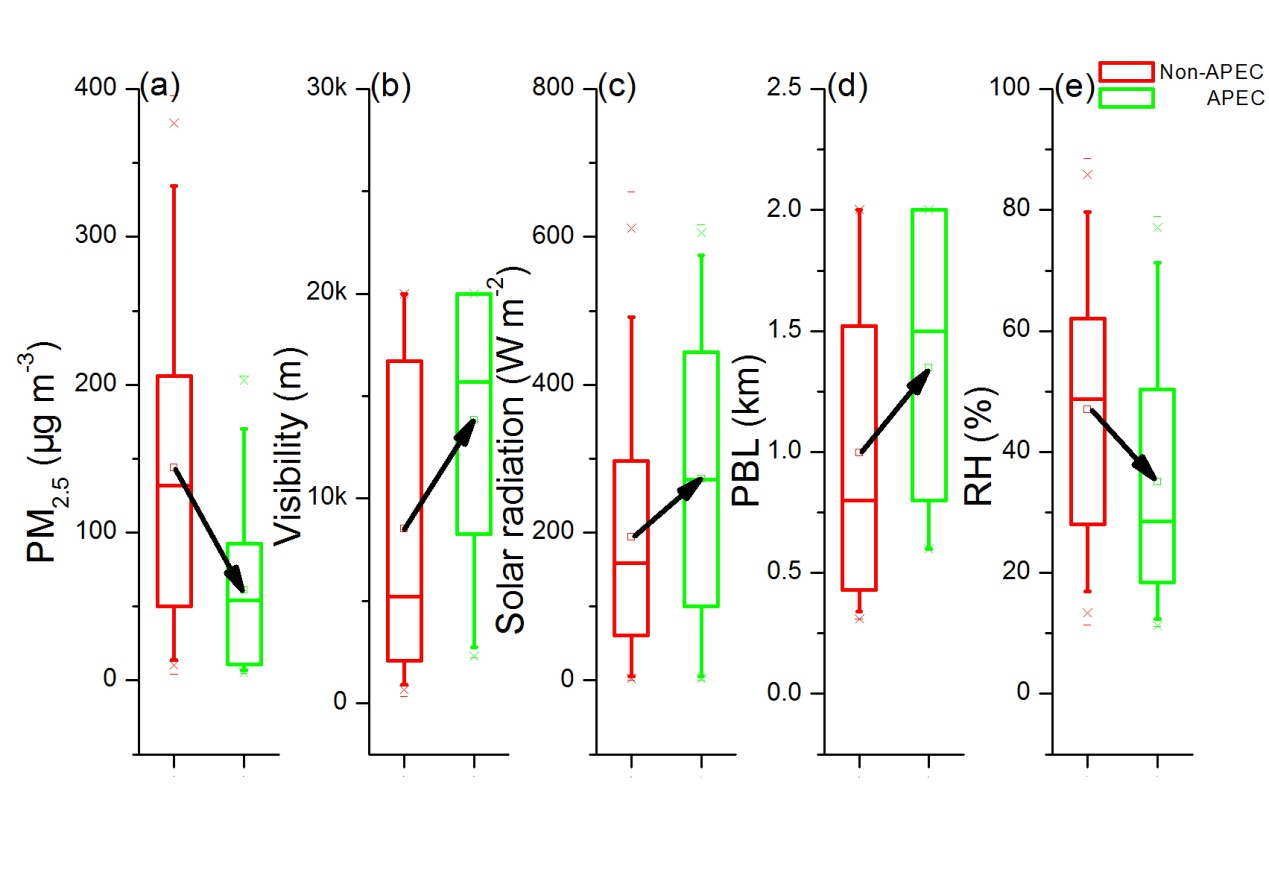


Figure S1. Comparison of observations between the APEC (red) and non-APEC (green) periods, including PM_2.5_ (a), visibility (b), RH (c), total solar radiation (d), and PBL height (e)


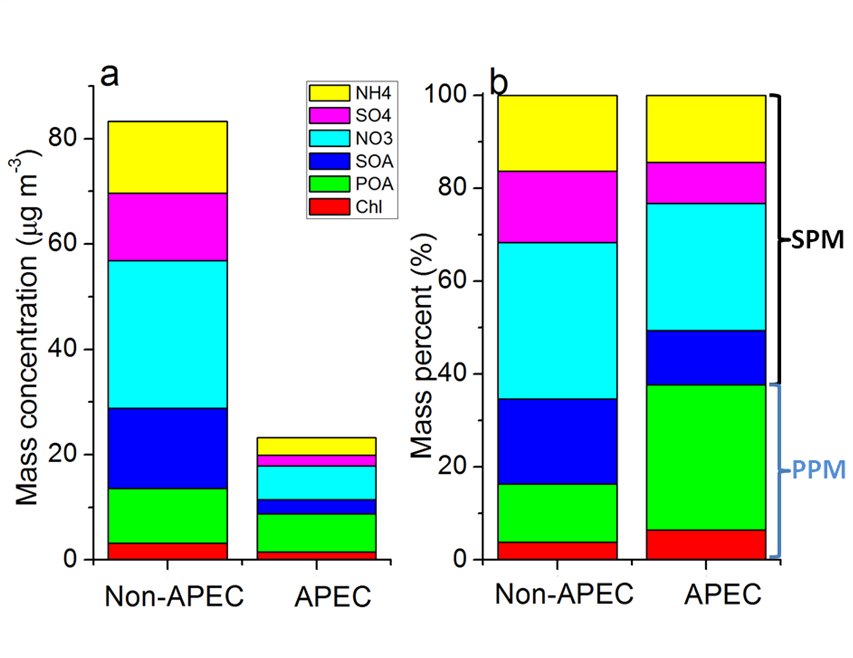


Figure S2. The measured primary particles (PPM), including Chl and POA, and secondary particles (SPM), including SO_4_, NO_3_, NH_4_, and SOA, under APEC and Non-APEC. The POA contains HOA, COA, and CCOA. The SOA is composed by OOA. The left panel presents the mass concentrations (μg/m^3^), and the right panel shows the mass percentage (%).

Figure S3. SW radiation, Temperature, relative humidity (RH), turbulence kinetic energy (TKE), PBL height (PBLH), and PM_2.5_ concentration over the study region under 4 scenarios. The PBLH is averaged during 13:00-15:00, SW is averaged during day time, and other variables are averaged during 18–21 December.
